# Graphene Schottky Junction on Pillar Patterned Silicon Substrate

**DOI:** 10.3390/nano9050659

**Published:** 2019-04-26

**Authors:** Giuseppe Luongo, Alessandro Grillo, Filippo Giubileo, Laura Iemmo, Mindaugas Lukosius, Carlos Alvarado Chavarin, Christian Wenger, Antonio Di Bartolomeo

**Affiliations:** 1 Physics Department “E. R. Caianiello”, University of Salerno, via Giovanni Paolo II n. 132, 84084 Fisciano, Italy; agrillo@unisa.it (A.G.); liemmo@unisa.it (L.I.); 2 CNR-SPIN Salerno, via Giovanni Paolo II n. 132, 84084 Fisciano, Italy; filippo.giubileo@spin.cnr.it; 3 IHP–Leibniz Institut fuer innovative Mikroelektronik, Im Technologiepark 25, 15236 Frankfurt (Oder), Germany; lukosius@ihp-microelectronics.com (M.L.); alvarado@ihp-microelectronics.com (C.A.C.); wenger@ihp-microelectronics.com (C.W.); 4 Brandenburg Medical School Theodor Fontane, 16816 Neuruppin, Germany; 5 Interdepartmental Centre NanoMates, University of Salerno, via Giovanni Paolo II n. 132, 84084 Fisciano, Italy

**Keywords:** graphene, Schottky barrier, diode, photodetector, heterojunction, MOS (Metal Oxide Semiconductor) capacitor, responsivity

## Abstract

A graphene/silicon junction with rectifying behaviour and remarkable photo-response was fabricated by transferring a graphene monolayer on a pillar-patterned Si substrate. The device forms a 0.11 eV Schottky barrier with 2.6 ideality factor at room temperature and exhibits strongly bias- and temperature-dependent reverse current. Below room temperature, the reverse current grows exponentially with the applied voltage because the pillar-enhanced electric field lowers the Schottky barrier. Conversely, at higher temperatures, the charge carrier thermal generation is dominant and the reverse current becomes weakly bias-dependent. A quasi-saturated reverse current is similarly observed at room temperature when the charge carriers are photogenerated under light exposure. The device shows photovoltaic effect with 0.7% power conversion efficiency and achieves 88 A/W photoresponsivity when used as photodetector.

## 1. Introduction

The discovery of two-dimensional (2D) materials such as graphene [1], MoS_2_ [2,3], WSe_2_ [4,5], phosphorene and so on [6], has attracted the interests of the scientific community in the recent years. Graphene is still one the most studied materials for its 2D honeycomb structure, high electron mobility, high electrical and thermal conduction, low optical absorption coefficient and easy fabrication methods [1,7,8]. Large graphene layers can be easily synthesized by chemical vapor deposition (CVD) and integrated into the existing semiconductor device technologies. These properties make graphene the perfect candidate to realize a new generation of transistors [9,10,11,12,13,14], diodes [15,16,17,18,19,20], chemical-biological sensors [21,22,23], photodetectors and solar cells [24,25,26,27,28,29,30]. In the recent years, a lot of activity has been focused on the graphene/silicon junction (gr/Si) as one of the simplest graphene devices offering the possibility to study the physical phenomena that occur at the interface between 2D and 3D materials [31]. The gr/Si junction usually forms a Schottky barrier and behaves as a rectifier with a current-voltage (I-V) characteristic similar to that of a metal/semiconductor Schottky diode [31,32]. Because of its particular band structure, graphene possesses low electron density of states close to the Dirac point, hence the Fermi level is highly dependent on charge transfer to or from it. In the gr/Si junction, the application of a bias affects the charge transfer process and the consequent shift of the graphene Fermi energy modulates the gr/Si Schottky barrier height, which becomes therefore bias dependent [31,32]. Indeed, adding such a feature into the standard thermionic emission (T.E.) theory provides an accurate model to describe the gr/Si experimental I-V characteristics [31,33]. Gr/Si Schottky diodes are characterized by a higher ideality factor (n>2) than metal/semiconductor devices (n∼1.3) [31]. The higher *n* arises because native oxide layers are generally formed at the interface during the graphene transfer process along with silicon interface trap states and/or metallic contamination [34,35]. Obviously, the ideality of the junction can be improved by reducing the interface defects, for instance through a suitable patterning of the substrate. Indeed, the gr/Si tip junctions that we presented in a previous work showed an ideality factor of 1.5 as the patterning of the Si substrate in a tip-array geometry reduces the probability of finding defects or contaminates at the junction, compared to a planar junction of the same area [17]. In addition to that, the tip geometry amplifies the electric field close to the junction, inducing a potential that shifts the graphene Fermi level even at low bias. We exploited such a feature to realize a bias-tunable graphene-based Schottky barrier device [17].

Modifying the substrate geometry is a viable approach to improve the gr/Si device performance or its photoresponse when used as a photodetector. We remark that the photoresponsivity of the gr/Si junction has been also improved by acting on the device structure. One possible way is to reduce the oxide layer underneath the graphene in order to create a metal/oxide/semiconductor (MOS) capacitor next to the gr/Si junction perimeter. Indeed, such an MOS capacitor plays an important role in the photo-charge collection process, by providing photogenerated carriers from the Si substrate to the junction [16,18,19,20,36].

In this work, we combine the tip geometry and the MOS capacitor approach, by fabricating a graphene/silicon junction on Si pillars to realize a bias-tunable Schottky diode that can be used also for photovoltaic and photodetection applications. The pillar perimeter works similarly to the nanotips in enhancing the electric field at the junction but is easier to fabricate and provides a better control of the MOS capacitor areas. We present an extensive analysis of the I-V characteristics of gr/Si pillar junction and evaluate the relevant parameters using the T.E. model and the Cheung and Cheung (C.C.) method [31,32,37]. We also investigate the photo response and the photovoltaic effect of the device using white LED light at different intensities.

## 2. Materials and Methods

**Figure 1:**
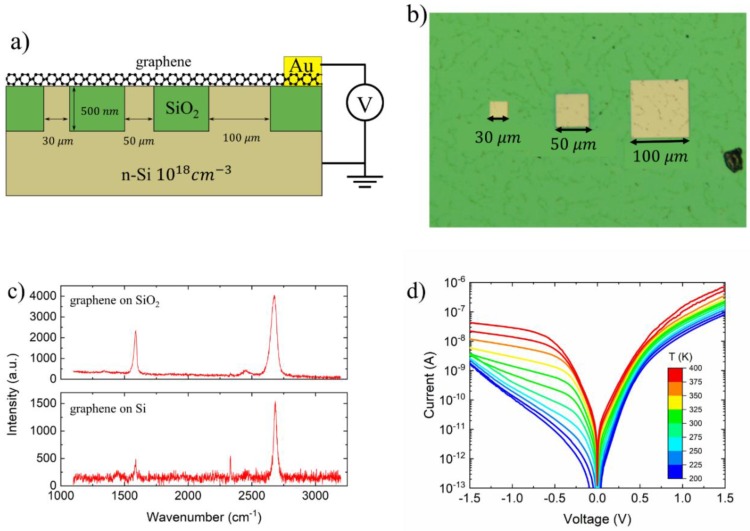
(**a**) Two-dimensional (2D) schematic view of the gr/Si-pillar device. (**b**) Optical microscope image of the pillars. (**c**) Raman spectroscopy of the graphene on SiO_2_ and Si. (**d**) The current-voltage (I-V) characteristic of the device measured from 200 K to 400 K.

Figure 1a shows the schematic view of the gr/Si-pillar junction. Starting from a highly n-doped silicon substrate $\left(∼10^{18}{\;\mathrm{cm}}^{-3}\right)$ three pillars with the height of ∼500 nm and square sections of 30 μm, 50 μm and 100 μm per side were patterned by photolithography (Figure 1b). In a gr/Si junction the Schottky barrier is controlled by the sharper geometries, that is by the pillar perimeter in our case. As the three pillars have similar perimeter/area ratio (∼10%), we expect that they contribute in a similar way to the junction properties. A SiO_2_ layer was CVD-deposited until it covered the silicon pillars. Chemical-mechanical polishing (CMP) was then used to remove the oxide layer on the top of the pillars. After that, a graphene layer was transferred from Cu foil on the pillars with a method detailed elsewhere [35].

The Raman spectrum of the graphene measured on the SiO_2_ and Si pillars is shown in Figure 1c. The plot shows two clear peaks at $∼1568\;{\mathrm{cm}}^{-1}$ and $∼2680\;{\mathrm{cm}}^{-1}$ which indicates that graphene is a good quality monolayer.

A gold contact (anode) was evaporated on the sample through a shadow mask. The other contact (cathode) was formed by coating silver paste on the scratched back-side of the Si substrate. The I-V measurements were performed with a Keithley Semiconductor Characterization System 4200 (SCS-4200) connected to a Janis probe station. During the measurements the sample was kept in dark and at a pressure of 1 mbar.

## 3. Results

Figure 1d shows the I-V characteristics measured for the gr/Si-pillar junction at different temperatures in the range 200–400 K. From low to room temperature the gr/Si junction shows an exponential reverse current which is typical of gr/Si junctions [17]. At higher temperatures, after the initial fast growth of the ohmic regime at low bias, the reverse current exhibits a gradual weaker dependence on the bias until it becomes quasi-saturated. The I-V characteristic at room temperature shows a rectification factor of two orders of magnitude at ±1.5 V.

The exponential reverse current growth at lower temperatures in Figure 1d can be explained considering the Fermi level shift due to the graphene low density of states, which reduces the Schottky barrier in reverse bias [31]. The variation of the barrier can be contributed also by the geometry and doping level of the substrate through the image-force barrier lowering. The pillar geometry magnifies the electric field around the perimeter where a wider depletion layer is created. Such a depletion layer is mirrored by charges in graphene, which cause an up-shift of the Fermi level and a reduction of the Schottky barrier. The high doping of the Si substrate can further contribute to barrier lowering through the image force effect. Conversely, the change of behaviour at higher temperatures indicates that the augmenting thermal generation rate in the depletion layer dominates the reverse leakage current which becomes less sensitive to the bias. The slight deviation of such current from saturation can be ascribed to image force barrier lowering [38,39].

To determine the Schottky diode parameters, we use the T.E. model with voltage dependent Schottky barrier height $q\phi_{B}$ [31], expressed by the equations:

(1) $$I=I_{0}e^{\frac{qV}{nkT}}\left(1-e^{-\frac{qV}{kT}}\right),$$

(2) $$I_{0}=AA^{*}T^{2}e^{-\frac{q\phi_{B}}{kT}},$$

where *I*_0_ is the reverse saturation current, *q* the electron charge, n>1 the ideality factor, k the Boltzmann constant, T the temperature, A the junction area, $A^{*}=\frac{4\pi m_{e}^{*}k^{2}}{h^{3}}=112{\;A\;\mathrm{cm}}^{-2}{\;K}^{-2}$ the Richardson constant for n-type Si ($m_{e}^{*}$ is the electron effective mass and h is the Plank constant) [40]. For qV>nkT, Equations (1) and (2) can be rewritten as:

(3) $$\ln\left(I\right)=\ln\left(I_{0}\right)+\frac{qV}{nkT}\;,$$

(4) $$\ln\left(\frac{I_{0}}{T^{2}}\right)=\ln\left(AA^{*}\right)-\frac{q\phi_{B}}{kT}.$$

**Figure 2:**
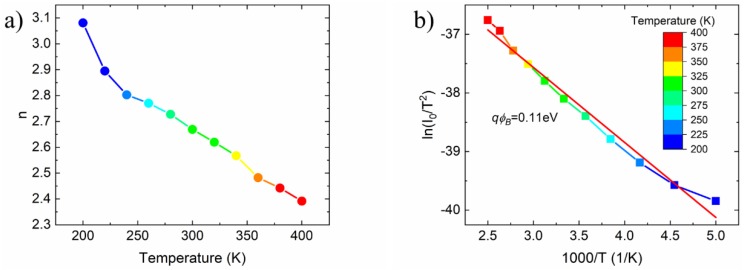
(**a**) Ideality factor vs the temperature extracted from the thermionic emission (T.E.) model (**b**) Richardson plot of the $\ln\left(I_{0}/T^{2}\right)$ versus $10^{3}/T$.

According to Equation (3), the straight-line fitting of the ln(I)-V characteristics for qV>>kT can be used to extrapolate the reverse current $I_{0}$ at zero bias and to estimate the ideality factor n. The so-obtained ideality factor as a function of temperature is shown in Figure 2a. The ideality factor at room temperature is n≈2.6 and is a monotonic decreasing function of the temperature because several non-idealities manifest more at lower temperatures. These non-idealities include metal residues consequence of the etching process (Cu in this case) which form carrier recombination centers, interface states at the junction which lead to charge trapping and detrapping, and the presence of a native oxide layer [31,34]. The zero-bias current, $I_{0}$, is used in the Richardson plot, $\ln\left(I_{0}/T^{2}\right)$ vs 1∕T, shown in Figure 2b, which, according to Equation (4), yields a Schottky barrier at zero-bias of 0.11 eV and $\ln\left(AA^{*}\;\right)=-33.72$. Since the effective gr/Si junction contact area is $∼\;1.34\cdot 10^{-2}\;{\mathrm{mm}}^{2}$, the Richardson constant is $A^{*}\;=\;1.68\cdot 10^{-9}\;{\mathrm{Acm}}^{2}K^{-2}$. A possible explanation for the low Richardson constant and the ideality factor *n* > 2 is the presence of a thin oxide layer [16]. Taking into account the native oxide thickness, Equation (2) can be modified by adding a tunnelling factor as:

(5) $$I_{0}=AA^{*}\exp\left(-\chi^{\frac{1}{2}}\delta \right)\exp-\left(\frac{q\phi_{B}}{kT}\right),$$

where δ (expressed in Å) is the oxide layer thickness and χ≈3 eV is the differences between the energy Fermi level and the conduction band minimum of SiO_2_. From Equation (5), we estimated an oxide layer of 15 Å, which is thin enough to allow a tunnelling current, but can sustain a voltage drop and affect the I-V characteristic of the junction.

At higher positive bias (V≳ 0.8 V), the thermionic emission current is limited by the series resistance $R_{S}$, which is the lump sum of contact, graphene and substrate resistances. By taking it into account, Equation (1) can be rewritten as

(6) $$I=I_{0}e^{\frac{q\left(V-IR_{S}\right)}{nkT}},$$

And from Equation (6), two new equations can be derived when $V-IR_{S}>nkT/q$ [37]:

(7) $$\frac{dV}{d\left(\ln\left(I\right)\right)}=IR_{S}-\frac{nkT}{q},$$

(8) $$H\left(I\right)=IR_{S}+n\phi_{B},$$

where H(I) is defined as:

(9) $$H\left(I\right)=V-\frac{nkT}{q}\ln\left(\frac{I}{AA^{*}T^{2}}\right).$$

**Figure 3:**
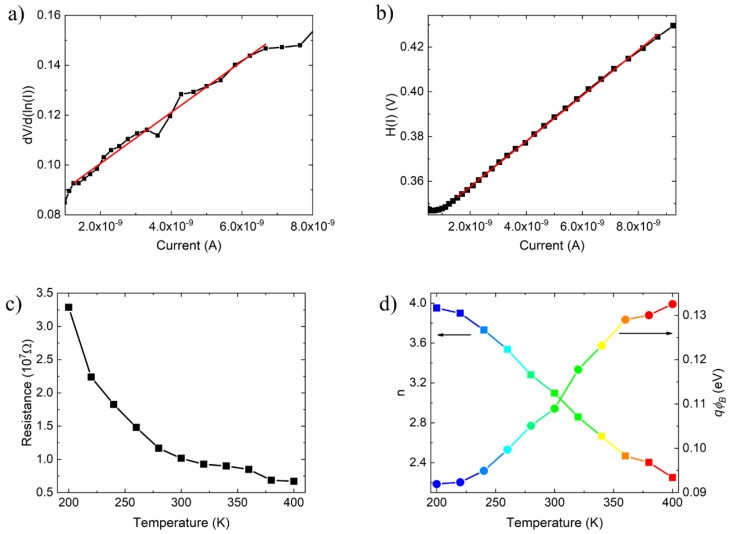
Cheung’s plot of (**a**) dV/dln(I) vs I and (**b**) H(I) vs I at 300K. (**c**) Devices series resistance, (**d**) ideality factor and the Schottky barrier extracted from the Cheung and Cheung (CC) method versus the temperature.

Accordingly, the series resistance and the ideality factor can be extrapolated from the slope and the intercept of the dV⁄d(ln(I)) vs I plot (Figure 3a), respectively, while the Schottky barrier can be estimated from the intercept of H(I) vs I plot (Figure 3b). Using this method, at room temperature, we obtain 10 MΩ series resistance and ideality factor ∼3. Figure 3c,d display the series resistance, the ideality factor and $q\phi_{B}$ measured at different temperatures. The decreasing series resistance with increasing temperature shows the typical semiconductor behaviour. This behaviour cannot be attributed to silicon, Au or Ag paste in this temperature range [41,42,43]. Therefore, it can only be caused by the graphene layer. The resistance drop at high temperature and the negative $dR_{S}/dT$ has been reported for both exfoliated and CVD grown graphene [44,45,46]. The graphene semimetal behaviour has been attributed mainly to the thermally activated transport through the inhomogeneous electron-hole puddles, the formation of which is favoured by the transfer process of CVD-grown graphene [35,46].

**Figure 4:**
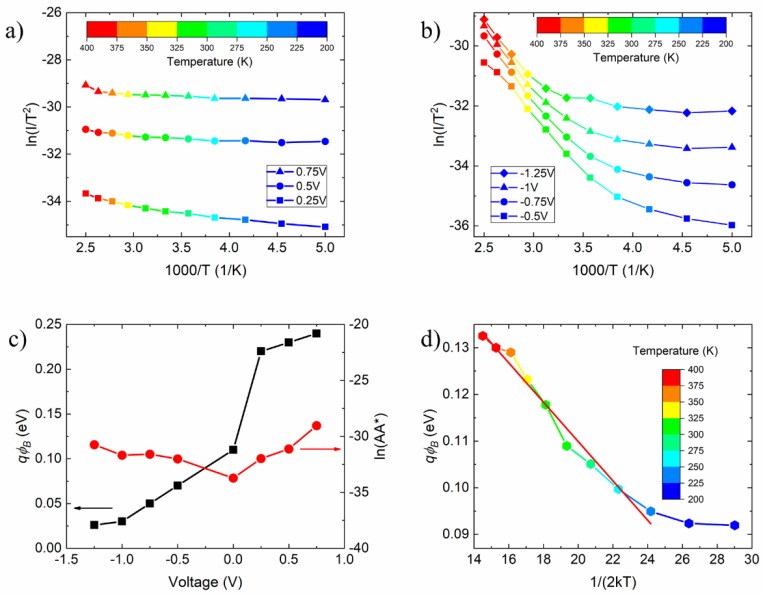
(**a**) Richardson plot of $\ln\left(I/T^{2}\right)$ vs $10^{3}\;T$ in forward and (**b**) in reverse bias. (**c**) Schottky barrier and $\ln\left(AA^{*}\right)$ respect the bias. (**d**) Schottky barrier height at zero bias as a function of temperature.

Using Equations (7)–(9), we estimate $q\phi_{B}$ at different temperatures (Figure 3d); in particular $q\phi_{B}\approx 0.11\;\mathrm{eV}$ at room temperature which is in agreement with the previous evaluation. The temperature growing $q\phi_{B}$ is an indication of possible spatial inhomogeneities. The homogeneity of the barrier will be discussed later. In Figure 4a,b we show the Richardson plot at given forward and reverse biases. In forward bias, the temperature dependence of the current has a linear behaviour, which is in agreement with the T.E. theory. Contrarily, in reverse bias, the evolving behaviour of the current, from exponential to saturation trend, is reflected in the Richardson plot (Figure 4b), which for *T* ≤ 300 K is similar to the forward bias one (Figure 4a), while at higher temperature shows rising converging curves. Because of this, we consider only the lower temperature part of the curves in Figure 4b (T ≤ 300 K) to determinate the Schottky barrier and the $\ln\left(AA^{*}\right)$, which are displayed in Figure 4c. We highlight that the Schottky barrier increases with the applied voltage, as expected. In forward bias, the graphene Fermi energy shifts down with respect to the semiconductor energy bands, thus increasing the Schottky barrier, while the opposite occurs in reverse bias. The relative shift, and therefore the barrier variation, is enhanced by the magnified electric field of the pillar and is made possible by the depinning of the Fermi level caused by the thin interfacial oxide layer [17,40].

Because of the CMP treatment (see Section 2), there is a possibility that the pillar top surface is not homogeneous and there could be points where the Schottky barrier is higher or lower. Following Refs. [17,40,47], we assume that the spatial variation of the Schottky barrier can be described by a Gaussian distribution. Therefore, the temperature dependence of the barrier is expressed as:

(10) $$q\phi_{B}=q\phi_{BM}-\frac{q\sigma^{2}}{2kT}\;,$$

where $q\phi_{BM}$ is the maximum Schottky barrier and σ is the standard deviation of the Gaussian distribution. σ characterizes the inhomogeneity of the Schottky barrier and can be extracted from a plot of $q\phi_{B}$ vs 1/2kT (Figure 4d). We obtain σ=45 meV, which is lower than those reported in literature for CVD grown graphene [48,49]. Since the graphene was CVD grown, the low standard variation can be considered as a remarkable advantage of the patterning of the substrate.

**Figure 5:**
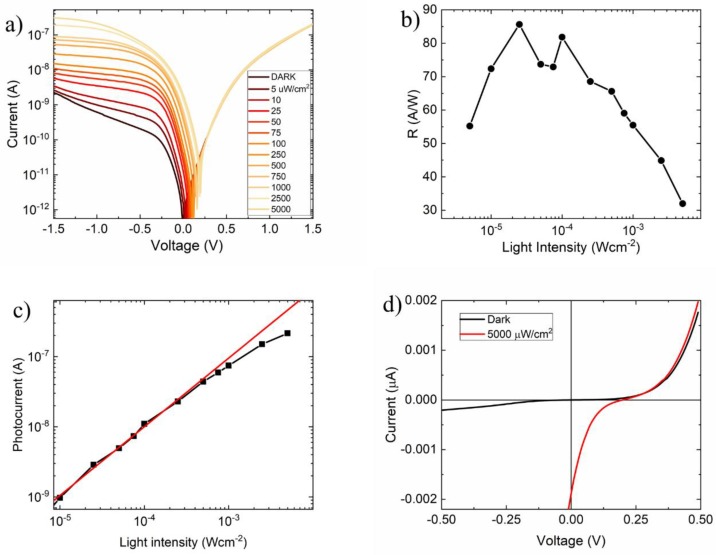
(**a**) I-V characteristic in semilogarithmic scale of the gr/Si pillar device measured at different intensity illumination level. (**b**) Responsivity of the gr/Si pillar device as function of the light intensity. (**c**) Photocurrent measured at −1V and at different light intensities in logarithmic scale. (**d**) I-V characteristic measured in dark (black line) and at 5 mWcm^−2^ (red line).

Finally, we measured the gr/Si response to light. Figure 5a shows the semi-logarithmic I-V curves of the device measured under different white LED light intensities. The responsivity $ℛ=\left(I_{light}-I_{dark}\right)/P_{opt}$ ($I_{light}$ and $I_{dark}$ are the current measured at −1V under illumination and in dark, respectively) as a function of the incident light power $P_{opt}$ is shown in Figure 5b. The device presents a responsivity with a maximum of ∼88 A/W at $10^{-5}-10^{-4}\;{\mathrm{Wcm}}^{-2}$, which decreases at higher intensities. The reduction of the responsivity at higher intensities is due to the raising recombination rate. Indeed, at high illumination, the increasing of electron-hole pair density in the depletion layer enhances the recombination rate thus making the photocurrent deviate from its linearly behaviour as shown Figure 5c.

Remarkably, the device achieves a reverse current that can be greater than the forward one. The high reverse current measured at high illumination confirms that there is a contribution to the junction current from the photogeneration occurring in the substrate areas where graphene forms a MOS capacitor with Si, as explained in previous works [16,18,36,50]. Furthermore, we note that the photogeneration has the same effect as the thermal generation in shaping the I-V curves of the device. Figure 5d shows the I-V measured in dark and under illumination at $5\;\mathrm{mW}/{\mathrm{cm}}^{2}$ in linear scale. A photovoltaic effect with an open circuit voltage around 0.19 V, which is close to the estimated Schottky barrier height, and a short circuit current of 1.8 nA, corresponding to ∼0.7% power conversion efficiency, can be clearly observed. The conversion efficiency can be improved by lowering the doping of the Si substrate, which would result in an extended depletion layer for enhanced light absorption, and by reducing the shunt and series resistance that would increase the cell fill factor.

## 4. Conclusions

In conclusion, we fabricated a gr/Si pillar junction that possesses both a bias-tunable Schottky barrier, remarkable photoresponse and appreciable power conversion efficiency. The reverse current grows exponentially with reverse bias at lower temperatures, while it shows a saturation at higher temperatures or under illumination. Such behaviour has been explained by taking into account the thermo- and photo- generated charges both at the gr/Si junction and in the surrounding regions.
